# More research is needed on how to prevent vaping among young people

**DOI:** 10.1371/journal.pmed.1004391

**Published:** 2024-04-15

**Authors:** Coral Gartner, Wayne D. Hall

**Affiliations:** 1 NHMRC Centre of Research Excellence on Achieving the Tobacco Endgame, School of Public Health, The University of Queensland, Herston, Australia; 2 National Centre for Youth Substance Use Research, The University of Queensland, St Lucia, Australia

We need effective policies to reduce youth use of ENDS globally. Countries have implemented a variety of regulations to reduce ENDS use by youth, including comprehensive sales bans, but more research is needed on the effectiveness of these policies.

Tobacco use continues to be the leading preventable cause of global disease burden [1]. However, concern is increasing about the growing use of other nicotine-containing products among youth, especially Electronic Nicotine Delivery Systems (ENDS), due to uncertainty about their long-term health risks [2] and whether they provide a “gateway” to tobacco smoking [3]. The World Health Organization (WHO) recommends that member states either ban or strictly regulate the sale of ENDS to prevent youth uptake [2]. Thirty-four countries have banned their sale [2].

A complete ban on sales may seem the most straightforward way to prevent young people from using ENDS (also known as vaping), but there is little evidence to support its effectiveness, in comparison to other policies in the MPOWER policy package that WHO recommends to reduce the global burden of tobacco smoking [2]. Only 8 (24%) out of the 34 countries that have banned the sale of ENDS have monitored the impact of the bans on ENDS use by youth and adults [2]. Recent analyses of survey data suggest that these bans have had little effect. A systematic review of survey data from 2014 to 2021 reported that 7.7% of young people aged under 26 years currently use ENDS [2], and rates in countries with a sales ban are no lower than in those without bans (e.g., 9.0% in Mexico and 6.7% in Thailand) [4].

Countries that have not banned ENDS have adopted a variety of approaches to regulate them. For example, England and New Zealand (also known as Aotearoa) allow ENDS to be sold as consumer products and encourage adults to use them for smoking cessation and as a lower risk alternative to cigarettes. The USA allows ENDS to be sold to consumers as a substitute for cigarettes, but US health authorities generally discourage their use [5]. Australia regulates ENDS as a prescription medicine, but no ENDS have been approved as cessation products, and leading medical organisations discourage doctors from prescribing them as unapproved medicines [5]. ENDS use among youth has increased in Australia and New Zealand despite their very different policy approaches. In New Zealand, daily ENDS use has increased among adolescents aged 14 to 15 years from around 1% in 2015 to 10.6% in 2022 [6]. In Australia, a recent survey suggests that ENDS use in the past year among 14- to 17-year-olds may be even higher than in countries with fewer restrictions on ENDS sales [7].

ENDS use is likely to be less harmful than continuing to smoke cigarettes [8], but there are compelling reasons to minimise uptake among youth who have not smoked tobacco. These include the risks of nicotine addiction and uncertainty about the health risks of long-term ENDS use [8]. In designing policies to minimise ENDS use among youth, however, regulators also need to take account of the evidence that ENDS can help adults to quit smoking [9]. Regulators also need to minimise potential adverse effects of sales bans [10], such as generating an illicit market for ENDS products if people who use them are unwilling to comply with a prescription system. Countries also should avoid imposing criminal penalties on adults who use ENDS or expelling adolescents from high school for vaping.

In Australia, the combination of a retail sales ban and prescription model has not prevented ENDS use among youth [10]. Australia is a high-income country, with well-resourced border services, but its law enforcement and health authorities have not been able to prevent large-scale illegal importation of ENDS or prevent the uptake of vaping by youth. Australian authorities have recently responded with new regulations that ban the importation of ENDS or ENNDS (electronic non-nicotine delivery systems) except by pharmacies with an import permit and that will ban the sale of ENNDS except via pharmacies and mandatory pharmaceutical-style packaging requirements [11]. Further restrictions on ENDS have also been announced for the UK to address youth use, including a ban on disposable ENDS, restrictions on flavours, ENDS packaging and product displays in retail settings. It will be some time before the effectiveness (and unintended effects) of these restrictions will be clear.

More rigorous research into the effectiveness of different ways of regulating ENDS is needed to produce more evidence-based policy. This will require the collection of good quality survey data on youth ENDS use to enable robust comparisons between the effects of different policies in different countries, such as the ITC Youth Tobacco and Vaping Survey and the Global Youth Tobacco Survey [10]. Increased investment in collecting standardised, reliable national survey data on youth and adult ENDS/ENNDS and tobacco use should accordingly be given a high global priority by the WHO.
